# Major Release of 161 Whole-Genome Sequences from the International *Pseudomonas* Consortium Database

**DOI:** 10.1128/MRA.00013-19

**Published:** 2019-03-28

**Authors:** Julie Jeukens, Jean-Guillaume Emond-Rheault, Luca Freschi, Irena Kukavica-Ibrulj, Roger C. Levesque

**Affiliations:** a Institut de biologie intégrative et des systèmes (IBIS), Université Laval, Québec, QC, Canada; University of Southern California

## Abstract

Pseudomonas aeruginosa is an environmental bacterium and opportunistic pathogen. Here, we present draft genome sequences for 161 isolates from diverse clinical and environmental sources. This set of genome sequences complements other major public data releases from the International *Pseudomonas* Consortium Database.

## Abstract (precis)

Pseudomonas aeruginosa is an environmental bacterium and opportunistic pathogen. Here, we present draft genome sequences for 161 isolates from diverse clinical and environmental sources.

## ANNOUNCEMENT

Over the past 3 years, the International Pseudomonas aeruginosa Consortium has reached its original goal of sequencing 1,000 Pseudomonas aeruginosa genomes for the study of genome evolution, antibiotic resistance, and virulence genes (1). This sequencing project relies on a large strain collection that was assembled with the aim of truly representing the worldwide P. aeruginosa population. Details of this collection are available through the International *Pseudomonas* Consortium Database (IPCD; http://ipcd.ibis.ulaval.ca/). It spans all continents, with a total of 1,723 isolates from cystic fibrosis patients, other types of human infections, animal infections, plants, soil, and water. The 1,030 genome sequences of the IPCD are now gradually becoming publicly available via a series of published studies (2–4; J. Jeukens, L. Freschi, I. Kukavica-Ibrulj, J.G. Emond-Rheault, C. Allard, J. Barbeau, A. Cantin, S.J. Charette, E. Déziel , F. Malouin, J. Milot, D. Nguyen, C. Popa, B. Boyle, R.C. Levesque, submitted for publication). Here, we present 161 new genome sequences representing isolates from various clinical and environmental sources that were not included in these studies.

Bacterial colonies were isolated on Difco *Pseudomonas* isolation agar (BD, Sparks, MD). Genomic DNA was extracted from overnight liquid cultures (37°C, LB medium) using the E-Z 96 tissue DNA kit (Omega Bio-tek, Norcross, GA). Genomic DNA (250 to 750 ng) was mechanically fragmented for 40 s using a Covaris M220 instrument (Covaris, Woburn MA) with default settings. Fragmented DNA was transferred to a PCR tube, and library synthesis was performed with the NEBNext Ultra II DNA library prep kit for Illumina (New England Biolabs, Ipswich, MA) according to the manufacturer’s instructions. TruSeq HT adapters (Illumina, San Diego, CA) were used to barcode the libraries, which were each sequenced in 1/48 of an Illumina MiSeq 300-bp paired-end run at the Plateforme d’Analyses Génomiques of the Institut de Biologie Intégrative et des Systèmes (IBIS; Université Laval, Québec, Canada). Each data set was assembled *de novo* with the A5 pipeline version A5-miseq 20140521 (5). Genome assemblies were annotated with the NCBI Prokaryotic Genome Annotation Pipeline.

The resulting genome assemblies range from 15 to 99 scaffolds, with an average *N*_50_ value of 484,265 bp and a median coverage of 31× (Table 1). Combined with the IPCD’s comprehensive strain collection, this set of 161 genomes and other data releases from the IPCD constitute a uniformly generated, high-quality data set that can be used for further genomic analysis, such as the identification of antimicrobial resistance and prognostic markers (6).

**TABLE 1:** Whole-genome data sequencing for 161 P. aeruginosa isolates from the International *Pseudomonas* Consortium Database

Isolate identifier	BioSample no.	GenBank accession no.	SRA run no.	Country	Environmental sample	No. of scaffolds	Size (Mb)	*N*_50_ (bp)	GC content (%)	Median coverage (×)	IPCD URL
229	SAMN10478413	RXFK00000000	SRR8612906	Australia	Yes	51	6.4897	357,215	66.3	31	https://ipcd.ibis.ulaval.ca/index/display-detail/id/229/criteria/IsolateID/dbfunction2/Y/
236	SAMN10478414	RXFJ00000000	SRR8612905	Australia	Yes	36	6.31904	665,346	66.5	33	https://ipcd.ibis.ulaval.ca/index/display-detail/id/236/criteria/IsolateID/dbfunction2/Y/
245	SAMN10478415	RXFI00000000	SRR8612908	Australia	Yes	33	6.46403	475,712	66.4	29	https://ipcd.ibis.ulaval.ca/index/display-detail/id/245/criteria/IsolateID/dbfunction2/Y/
246	SAMN10478416	RXFH00000000	SRR8612907	Australia	Yes	35	6.30793	524,351	66.5	37	https://ipcd.ibis.ulaval.ca/index/display-detail/id/246/criteria/IsolateID/dbfunction2/Y/
247	SAMN10478417	RXFG00000000	SRR8612902	Australia	Yes	29	6.25049	548,266	66.6	30	https://ipcd.ibis.ulaval.ca/index/display-detail/id/247/criteria/IsolateID/dbfunction2/Y/
249	SAMN10478418	RXFF00000000	SRR8612901	Australia	Yes	28	6.39554	820,044	66.4	29	https://ipcd.ibis.ulaval.ca/index/display-detail/id/249/criteria/IsolateID/dbfunction2/Y/
252	SAMN10478419	RXFE00000000	SRR8612904	Australia	Yes	46	6.23503	403,875	66.4	30	https://ipcd.ibis.ulaval.ca/index/display-detail/id/252/criteria/IsolateID/dbfunction2/Y/
253	SAMN10478420	RXFD00000000	SRR8612903	Australia	Yes	44	6.50779	421,957	66.5	28	https://ipcd.ibis.ulaval.ca/index/display-detail/id/253/criteria/IsolateID/dbfunction2/Y/
270	SAMN10478421	RXFC00000000	SRR8612900	Australia	Yes	27	6.37643	714,696	66.5	25	https://ipcd.ibis.ulaval.ca/index/display-detail/id/270/criteria/IsolateID/dbfunction2/Y/
287	SAMN10478422	RXFB00000000	SRR8612899	Australia	Yes	42	6.56496	476,708	66.3	26	https://ipcd.ibis.ulaval.ca/index/display-detail/id/287/criteria/IsolateID/dbfunction2/Y/
317	SAMN10478423	RXFA00000000	SRR8612849	Australia	Yes	25	6.23387	772,847	66.6	31	https://ipcd.ibis.ulaval.ca/index/display-detail/id/317/criteria/IsolateID/dbfunction2/Y/
324	SAMN10478424	RXEZ00000000	SRR8612848	Australia	Yes	42	6.74979	341,256	66.1	27	https://ipcd.ibis.ulaval.ca/index/display-detail/id/324/criteria/IsolateID/dbfunction2/Y/
325	SAMN10478425	RXEY00000000	SRR8612847	Australia	Yes	46	6.82269	579,571	65.9	30	https://ipcd.ibis.ulaval.ca/index/display-detail/id/325/criteria/IsolateID/dbfunction2/Y/
331	SAMN10478426	RXEX00000000	SRR8612846	Australia	Yes	57	7.14726	278,589	65.8	32	https://ipcd.ibis.ulaval.ca/index/display-detail/id/331/criteria/IsolateID/dbfunction2/Y/
357	SAMN10478427	RXEW00000000	SRR8612845	Australia	Yes	47	6.44814	427,325	66.3	37	https://ipcd.ibis.ulaval.ca/index/display-detail/id/357/criteria/IsolateID/dbfunction2/Y/
367	SAMN10478429	RXEV00000000	SRR8612844	Australia	Yes	58	6.6265	408,679	66.1	32	https://ipcd.ibis.ulaval.ca/index/display-detail/id/367/criteria/IsolateID/dbfunction2/Y/
369	SAMN10478430	RXEU00000000	SRR8612843	Australia	Yes	37	6.98641	429,963	65.5	33	https://ipcd.ibis.ulaval.ca/index/display-detail/id/369/criteria/IsolateID/dbfunction2/Y/
371	SAMN10478431	RXET00000000	SRR8612842	Australia	Yes	27	6.46627	855,026	66.3	34	https://ipcd.ibis.ulaval.ca/index/display-detail/id/371/criteria/IsolateID/dbfunction2/Y/
377	SAMN10478432	RXES00000000	SRR8612841	Australia	Yes	28	6.3506	665,914	66.4	40	https://ipcd.ibis.ulaval.ca/index/display-detail/id/377/criteria/IsolateID/dbfunction2/Y/
380	SAMN10478433	RXER00000000	SRR8612840	Australia	Yes	48	7.12073	424,139	65.3	32	https://ipcd.ibis.ulaval.ca/index/display-detail/id/380/criteria/IsolateID/dbfunction2/Y/
381	SAMN10478434	RXEQ00000000	SRR8612782	Australia	Yes	42	6.35667	313,467	66.4	42	https://ipcd.ibis.ulaval.ca/index/display-detail/id/381/criteria/IsolateID/dbfunction2/Y/
382	SAMN10478435	RXEP00000000	SRR8612783	Australia	Yes	31	6.55857	788,768	66.3	33	https://ipcd.ibis.ulaval.ca/index/display-detail/id/382/criteria/IsolateID/dbfunction2/Y/
392	SAMN10478436	RXEO00000000	SRR8612780	Australia	Yes	31	6.34732	527,001	66.5	45	https://ipcd.ibis.ulaval.ca/index/display-detail/id/392/criteria/IsolateID/dbfunction2/Y/
394	SAMN10478437	RXEN00000000	SRR8612781	Australia	Yes	33	6.36336	522,219	66.4	37	https://ipcd.ibis.ulaval.ca/index/display-detail/id/394/criteria/IsolateID/dbfunction2/Y/
395	SAMN10478438	RXEM00000000	SRR8612778	Australia	Yes	46	6.30084	396,687	66.5	40	https://ipcd.ibis.ulaval.ca/index/display-detail/id/395/criteria/IsolateID/dbfunction2/Y/
396	SAMN10478439	RXEL00000000	SRR8612779	Australia	Yes	31	6.26785	501,476	66.6	44	https://ipcd.ibis.ulaval.ca/index/display-detail/id/396/criteria/IsolateID/dbfunction2/Y/
397	SAMN10478440	RXEK00000000	SRR8612776	Australia	Yes	34	6.40942	574,088	66.5	37	https://ipcd.ibis.ulaval.ca/index/display-detail/id/397/criteria/IsolateID/dbfunction2/Y/
411	SAMN10478441	RXEJ00000000	SRR8612777	Australia	Yes	26	6.45449	742,570	66.4	36	https://ipcd.ibis.ulaval.ca/index/display-detail/id/411/criteria/IsolateID/dbfunction2/Y/
412	SAMN10478442	RXEI00000000	SRR8612784	Australia	Yes	35	6.44969	472,767	66.3	60	https://ipcd.ibis.ulaval.ca/index/display-detail/id/412/criteria/IsolateID/dbfunction2/Y/
413	SAMN10478443	RXEH00000000	SRR8612785	Australia	Yes	26	6.33649	566,005	66.5	33	https://ipcd.ibis.ulaval.ca/index/display-detail/id/413/criteria/IsolateID/dbfunction2/Y/
414	SAMN10478444	RXEG00000000	SRR8612884	Australia	Yes	17	6.32485	803,605	66.5	36	https://ipcd.ibis.ulaval.ca/index/display-detail/id/414/criteria/IsolateID/dbfunction2/Y/
415	SAMN10478445	RXEF00000000	SRR8612883	Australia	Yes	30	6.35064	489,232	66.5	31	https://ipcd.ibis.ulaval.ca/index/display-detail/id/415/criteria/IsolateID/dbfunction2/Y/
432	SAMN10478446	RXEE00000000	SRR8612886	Australia	Yes	18	6.31667	599,470	66.6	30	https://ipcd.ibis.ulaval.ca/index/display-detail/id/432/criteria/IsolateID/dbfunction2/Y/
443	SAMN10478447	RXED00000000	SRR8612885	Australia	Yes	62	6.65026	225,319	66.3	45	https://ipcd.ibis.ulaval.ca/index/display-detail/id/443/criteria/IsolateID/dbfunction2/Y/
444	SAMN10478448	RXEC00000000	SRR8612880	Australia	Yes	35	6.48943	514,739	66.3	51	https://ipcd.ibis.ulaval.ca/index/display-detail/id/444/criteria/IsolateID/dbfunction2/Y/
445	SAMN10478449	RXEB00000000	SRR8612879	Australia	Yes	26	6.32336	1,034,629	66.5	58	https://ipcd.ibis.ulaval.ca/index/display-detail/id/445/criteria/IsolateID/dbfunction2/Y/
447	SAMN10478450	RXEA00000000	SRR8612882	Australia	Yes	33	6.3544	769,793	66.5	50	https://ipcd.ibis.ulaval.ca/index/display-detail/id/447/criteria/IsolateID/dbfunction2/Y/
448	SAMN10478451	RXDZ00000000	SRR8612881	Australia	Yes	52	7.09373	245,962	65.8	42	https://ipcd.ibis.ulaval.ca/index/display-detail/id/448/criteria/IsolateID/dbfunction2/Y/
449	SAMN10478452	RXDY00000000	SRR8612890	Australia	Yes	30	6.42484	594,857	66.4	60	https://ipcd.ibis.ulaval.ca/index/display-detail/id/449/criteria/IsolateID/dbfunction2/Y/
450	SAMN10478453	RXDX00000000	SRR8612889	Australia	Yes	28	6.27342	667,621	66.5	37	https://ipcd.ibis.ulaval.ca/index/display-detail/id/450/criteria/IsolateID/dbfunction2/Y/
451	SAMN10478454	RXDW00000000	SRR8612817	Australia	Yes	61	6.41992	270,976	66.4	30	https://ipcd.ibis.ulaval.ca/index/display-detail/id/451/criteria/IsolateID/dbfunction2/Y/
479	SAMN10478455	RXDV00000000	SRR8612818	Australia	No	52	7.03642	324,049	66	24	https://ipcd.ibis.ulaval.ca/index/display-detail/id/479/criteria/IsolateID/dbfunction2/Y/
481	SAMN10478456	RXDU00000000	SRR8612819	Australia	No	49	6.97704	343,147	66	27	https://ipcd.ibis.ulaval.ca/index/display-detail/id/481/criteria/IsolateID/dbfunction2/Y/
482	SAMN10478457	RXDT00000000	SRR8612820	Australia	No	70	7.06873	291,285	65.9	25	https://ipcd.ibis.ulaval.ca/index/display-detail/id/482/criteria/IsolateID/dbfunction2/Y/
517	SAMN10478458	RXDS00000000	SRR8612813	Australia	No	41	6.15601	488,545	66.6	37	https://ipcd.ibis.ulaval.ca/index/display-detail/id/517/criteria/IsolateID/dbfunction2/Y/
532	SAMN10478459	RXDR00000000	SRR8612814	Australia	No	38	6.2302	485,479	66.6	36	https://ipcd.ibis.ulaval.ca/index/display-detail/id/532/criteria/IsolateID/dbfunction2/Y/
540	SAMN10478460	RXDQ00000000	SRR8612815	USA	No	34	6.4579	517,479	66.4	28	https://ipcd.ibis.ulaval.ca/index/display-detail/id/540/criteria/IsolateID/dbfunction2/Y/
541	SAMN10478461	RXDP00000000	SRR8612816	USA	No	43	6.4603	482,685	66.4	26	https://ipcd.ibis.ulaval.ca/index/display-detail/id/541/criteria/IsolateID/dbfunction2/Y/
542	SAMN10478462	RXDO00000000	SRR8612811	USA	No	35	6.45359	454,597	66.4	23	https://ipcd.ibis.ulaval.ca/index/display-detail/id/542/criteria/IsolateID/dbfunction2/Y/
545	SAMN10478463	RXDN00000000	SRR8612812	USA	No	30	6.31901	778,034	66.5	36	https://ipcd.ibis.ulaval.ca/index/display-detail/id/545/criteria/IsolateID/dbfunction2/Y/
546	SAMN10478464	RXDM00000000	SRR8612916	USA	No	36	6.37277	777,972	66.5	29	https://ipcd.ibis.ulaval.ca/index/display-detail/id/546/criteria/IsolateID/dbfunction2/Y/
548	SAMN10478465	RXDL00000000	SRR8612915	USA	No	88	6.70739	249,180	66.3	21	https://ipcd.ibis.ulaval.ca/index/display-detail/id/548/criteria/IsolateID/dbfunction2/Y/
553	SAMN10478466	RXDK00000000	SRR8612914	USA	No	32	6.76989	438,875	66.1	28	https://ipcd.ibis.ulaval.ca/index/display-detail/id/553/criteria/IsolateID/dbfunction2/Y/
554	SAMN10478467	RXDJ00000000	SRR8612913	USA	No	31	6.77032	443,905	66.1	27	https://ipcd.ibis.ulaval.ca/index/display-detail/id/554/criteria/IsolateID/dbfunction2/Y/
561	SAMN10478468	RXDI00000000	SRR8612920	USA	No	19	6.44922	777,255	66.4	25	https://ipcd.ibis.ulaval.ca/index/display-detail/id/561/criteria/IsolateID/dbfunction2/Y/
562	SAMN10478469	RXDH00000000	SRR8612919	USA	No	21	6.44997	777,256	66.4	30	https://ipcd.ibis.ulaval.ca/index/display-detail/id/562/criteria/IsolateID/dbfunction2/Y/
564	SAMN10478470	RXDG00000000	SRR8612918	USA	No	44	6.39778	342,464	66.4	23	https://ipcd.ibis.ulaval.ca/index/display-detail/id/564/criteria/IsolateID/dbfunction2/Y/
566	SAMN10478471	RXDF00000000	SRR8612917	USA	No	41	6.96278	502,782	66	22	https://ipcd.ibis.ulaval.ca/index/display-detail/id/566/criteria/IsolateID/dbfunction2/Y/
748	SAMN10478472	RXDE00000000	SRR8612922	Japan	Yes	55	6.96666	426,725	66	32	https://ipcd.ibis.ulaval.ca/index/display-detail/id/748/criteria/IsolateID/dbfunction2/Y/
750	SAMN10478473	RXDD00000000	SRR8612921	Japan	Yes	40	6.63624	380,742	66.2	31	https://ipcd.ibis.ulaval.ca/index/display-detail/id/750/criteria/IsolateID/dbfunction2/Y/
751	SAMN10478474	RXFL00000000	SRR8612925	Japan	Yes	44	6.94121	367,696	66	29	https://ipcd.ibis.ulaval.ca/index/display-detail/id/751/criteria/IsolateID/dbfunction2/Y/
754	SAMN10478475	RXDC00000000	SRR8612926	Japan	Yes	42	6.71812	468,104	66.3	29	https://ipcd.ibis.ulaval.ca/index/display-detail/id/754/criteria/IsolateID/dbfunction2/Y/
755	SAMN10478476	RXDB00000000	SRR8612923	Japan	Yes	42	6.80593	554,454	65.9	26	https://ipcd.ibis.ulaval.ca/index/display-detail/id/755/criteria/IsolateID/dbfunction2/Y/
758	SAMN10478477	RXDA00000000	SRR8612924	Japan	Yes	67	6.8055	255,461	66.1	28	https://ipcd.ibis.ulaval.ca/index/display-detail/id/758/criteria/IsolateID/dbfunction2/Y/
759	SAMN10478478	RXCZ00000000	SRR8612929	Japan	Yes	68	6.91414	208,892	66	29	https://ipcd.ibis.ulaval.ca/index/display-detail/id/759/criteria/IsolateID/dbfunction2/Y/
760	SAMN10478479	RXCY00000000	SRR8612930	Japan	Yes	90	6.97551	203,554	66	24	https://ipcd.ibis.ulaval.ca/index/display-detail/id/760/criteria/IsolateID/dbfunction2/Y/
761	SAMN10478480	RXCX00000000	SRR8612927	Japan	Yes	81	6.81359	230,545	66.1	27	https://ipcd.ibis.ulaval.ca/index/display-detail/id/761/criteria/IsolateID/dbfunction2/Y/
762	SAMN10478481	RXCW00000000	SRR8612928	Japan	Yes	55	7.13133	257,983	65.7	25	https://ipcd.ibis.ulaval.ca/index/display-detail/id/762/criteria/IsolateID/dbfunction2/Y/
824	SAMN10478482	RXCV00000000	SRR8612888	Belgium	Yes	32	6.76679	515,774	66.1	34	https://ipcd.ibis.ulaval.ca/index/display-detail/id/824/criteria/IsolateID/dbfunction2/Y/
825	SAMN10478483	RXCU00000000	SRR8612911	Belgium	Yes	23	6.50556	515,348	66.4	28	https://ipcd.ibis.ulaval.ca/index/display-detail/id/825/criteria/IsolateID/dbfunction2/Y/
826	SAMN10478484	RXCT00000000	SRR8612798	Belgium	Yes	44	6.7263	401,909	66.2	31	https://ipcd.ibis.ulaval.ca/index/display-detail/id/826/criteria/IsolateID/dbfunction2/Y/
828	SAMN10478485	RXCS00000000	SRR8612797	Belgium	Yes	52	6.87521	348,379	65.9	29	https://ipcd.ibis.ulaval.ca/index/display-detail/id/828/criteria/IsolateID/dbfunction2/Y/
829	SAMN10478486	RXCR00000000	SRR8612801	Belgium	Yes	41	6.6716	366,373	66.3	28	https://ipcd.ibis.ulaval.ca/index/display-detail/id/829/criteria/IsolateID/dbfunction2/Y/
831	SAMN10478487	RXCQ00000000	SRR8612823	Belgium	Yes	27	6.78081	783,488	66.2	28	https://ipcd.ibis.ulaval.ca/index/display-detail/id/831/criteria/IsolateID/dbfunction2/Y/
1105	SAMN10478488	RXCP00000000	SRR8612810	Thailand	No	52	6.88085	480,584	66	27	https://ipcd.ibis.ulaval.ca/index/display-detail/id/1105/criteria/IsolateID/dbfunction2/Y/
1106	SAMN10478489	RXCO00000000	SRR8612851	Thailand	No	47	6.27294	385,450	66.5	30	https://ipcd.ibis.ulaval.ca/index/display-detail/id/1106/criteria/IsolateID/dbfunction2/Y/
1108	SAMN10478490	RXCN00000000	SRR8612796	Thailand	No	20	6.25092	765,594	66.5	31	https://ipcd.ibis.ulaval.ca/index/display-detail/id/1108/criteria/IsolateID/dbfunction2/Y/
1110	SAMN10478491	RXCM00000000	SRR8612865	Thailand	No	36	6.67589	608,561	66.2	30	https://ipcd.ibis.ulaval.ca/index/display-detail/id/1110/criteria/IsolateID/dbfunction2/Y/
1111	SAMN10478492	RXCL00000000	SRR8612827	Thailand	No	32	6.33641	439,305	66.4	34	https://ipcd.ibis.ulaval.ca/index/display-detail/id/1111/criteria/IsolateID/dbfunction2/Y/
1115	SAMN10478493	RXCK00000000	SRR8612826	Thailand	No	46	6.36923	287,370	66.4	33	https://ipcd.ibis.ulaval.ca/index/display-detail/id/1115/criteria/IsolateID/dbfunction2/Y/
1126	SAMN10478494	RXCJ00000000	SRR8612822	Thailand	No	35	6.39825	426,159	66.5	36	https://ipcd.ibis.ulaval.ca/index/display-detail/id/1126/criteria/IsolateID/dbfunction2/Y/
1130	SAMN10478495	RXCI00000000	SRR8612824	Thailand	No	39	6.99363	355,083	66	31	https://ipcd.ibis.ulaval.ca/index/display-detail/id/1130/criteria/IsolateID/dbfunction2/Y/
1132	SAMN10478496	RXCH00000000	SRR8612909	Thailand	No	27	6.58261	478,039	66.3	29	https://ipcd.ibis.ulaval.ca/index/display-detail/id/1132/criteria/IsolateID/dbfunction2/Y/
1134	SAMN10478497	RXCG00000000	SRR8612830	Thailand	No	36	6.40832	472,459	66.3	30	https://ipcd.ibis.ulaval.ca/index/display-detail/id/1134/criteria/IsolateID/dbfunction2/Y/
1135	SAMN10478498	RXCF00000000	SRR8612825	Thailand	No	34	6.93776	583,246	65.9	27	https://ipcd.ibis.ulaval.ca/index/display-detail/id/1135/criteria/IsolateID/dbfunction2/Y/
1136	SAMN10478499	RXCE00000000	SRR8612839	Thailand	No	40	6.47484	354,473	66.3	33	https://ipcd.ibis.ulaval.ca/index/display-detail/id/1136/criteria/IsolateID/dbfunction2/Y/
1146	SAMN10478500	RXCD00000000	SRR8612852	Thailand	No	45	6.73357	477,284	66	33	https://ipcd.ibis.ulaval.ca/index/display-detail/id/1146/criteria/IsolateID/dbfunction2/Y/
1150	SAMN10478501	RXCC00000000	SRR8612864	Thailand	No	38	6.27398	564,768	66.6	28	https://ipcd.ibis.ulaval.ca/index/display-detail/id/1150/criteria/IsolateID/dbfunction2/Y/
1175	SAMN10478502	RXCB00000000	SRR8612771	USA	Yes	35	6.48184	494,753	66.3	31	https://ipcd.ibis.ulaval.ca/index/display-detail/id/1175/criteria/IsolateID/dbfunction2/Y/
1178	SAMN10478503	RXCA00000000	SRR8612772	USA	Yes	15	6.45214	826,302	66.4	32	https://ipcd.ibis.ulaval.ca/index/display-detail/id/1178/criteria/IsolateID/dbfunction2/Y/
1179	SAMN10478504	RXBZ00000000	SRR8612891	USA	Yes	19	6.45026	664,784	66.4	38	https://ipcd.ibis.ulaval.ca/index/display-detail/id/1179/criteria/IsolateID/dbfunction2/Y/
1218	SAMN10478505	RXBY00000000	SRR8612887	Japan	Yes	33	6.904	563,468	65.9	34	https://ipcd.ibis.ulaval.ca/index/display-detail/id/1218/criteria/IsolateID/dbfunction2/Y/
1220	SAMN10478506	RXBX00000000	SRR8612912	Japan	Yes	36	6.48836	683,844	66.4	34	https://ipcd.ibis.ulaval.ca/index/display-detail/id/1220/criteria/IsolateID/dbfunction2/Y/
1222	SAMN10478507	RXBW00000000	SRR8612876	Japan	Yes	53	6.84601	368,499	65.9	30	https://ipcd.ibis.ulaval.ca/index/display-detail/id/1222/criteria/IsolateID/dbfunction2/Y/
1224	SAMN10478508	RXBV00000000	SRR8612821	Japan	Yes	50	6.96144	627,011	65.9	31	https://ipcd.ibis.ulaval.ca/index/display-detail/id/1224/criteria/IsolateID/dbfunction2/Y/
1226	SAMN10478509	RXBU00000000	SRR8612850	Japan	Yes	26	6.7744	757,878	66.2	29	https://ipcd.ibis.ulaval.ca/index/display-detail/id/1226/criteria/IsolateID/dbfunction2/Y/
1228	SAMN10478510	RXBT00000000	SRR8612867	Japan	Yes	53	6.50199	339,692	66.2	30	https://ipcd.ibis.ulaval.ca/index/display-detail/id/1228/criteria/IsolateID/dbfunction2/Y/
1230	SAMN10478511	RXBS00000000	SRR8612866	Japan	Yes	39	6.47734	314,052	66.3	31	https://ipcd.ibis.ulaval.ca/index/display-detail/id/1230/criteria/IsolateID/dbfunction2/Y/
1232	SAMN10478512	RXBR00000000	SRR8612775	Japan	Yes	33	6.51199	426,899	66.4	34	https://ipcd.ibis.ulaval.ca/index/display-detail/id/1232/criteria/IsolateID/dbfunction2/Y/
1234	SAMN10478513	RXBQ00000000	SRR8612863	Japan	Yes	55	6.51997	257,501	66.3	42	https://ipcd.ibis.ulaval.ca/index/display-detail/id/1234/criteria/IsolateID/dbfunction2/Y/
1236	SAMN10478514	RXBP00000000	SRR8612874	Japan	Yes	74	6.94925	340,243	66.2	35	https://ipcd.ibis.ulaval.ca/index/display-detail/id/1236/criteria/IsolateID/dbfunction2/Y/
1240	SAMN10478515	RXBO00000000	SRR8612875	Japan	Yes	76	6.93936	228,401	66	41	https://ipcd.ibis.ulaval.ca/index/display-detail/id/1240/criteria/IsolateID/dbfunction2/Y/
1356	SAMN10478516	RXBN00000000	SRR8612872	Germany	No	66	6.35478	233,990	66.4	18	https://ipcd.ibis.ulaval.ca/index/display-detail/id/1356/criteria/IsolateID/dbfunction2/Y/
1360	SAMN10478518	RXBM00000000	SRR8612873	Israel	No	23	6.27396	435,860	66.5	32	https://ipcd.ibis.ulaval.ca/index/display-detail/id/1360/criteria/IsolateID/dbfunction2/Y/
1361	SAMN10478519	RXBL00000000	SRR8612870	Germany	No	54	6.90503	383,627	66	23	https://ipcd.ibis.ulaval.ca/index/display-detail/id/1361/criteria/IsolateID/dbfunction2/Y/
1362	SAMN10478520	RXBK00000000	SRR8612871	Germany	No	28	6.29155	457,259	66.6	33	https://ipcd.ibis.ulaval.ca/index/display-detail/id/1362/criteria/IsolateID/dbfunction2/Y/
1363	SAMN10478521	RXBJ00000000	SRR8612868	Germany	No	46	6.74808	355,186	66	46	https://ipcd.ibis.ulaval.ca/index/display-detail/id/1363/criteria/IsolateID/dbfunction2/Y/
1364	SAMN10478522	RXBI00000000	SRR8612869	Germany	No	23	6.37015	771,128	66.5	35	https://ipcd.ibis.ulaval.ca/index/display-detail/id/1364/criteria/IsolateID/dbfunction2/Y/
1365	SAMN10478523	RXBH00000000	SRR8612877	Germany	No	62	6.74733	329,843	66	23	https://ipcd.ibis.ulaval.ca/index/display-detail/id/1365/criteria/IsolateID/dbfunction2/Y/
1366	SAMN10478524	RXBG00000000	SRR8612878	Germany	No	33	6.47395	477,720	66.4	30	https://ipcd.ibis.ulaval.ca/index/display-detail/id/1366/criteria/IsolateID/dbfunction2/Y/
1367	SAMN10478525	RXBF00000000	SRR8612897	Germany	No	64	6.36843	394,253	66.3	23	https://ipcd.ibis.ulaval.ca/index/display-detail/id/1367/criteria/IsolateID/dbfunction2/Y/
1369	SAMN10478526	RXBE00000000	SRR8612896	Germany	No	42	6.36112	439,762	66.4	42	https://ipcd.ibis.ulaval.ca/index/display-detail/id/1369/criteria/IsolateID/dbfunction2/Y/
1370	SAMN10478527	RXBD00000000	SRR8612770	Germany	No	99	7.22394	209,392	65.6	31	https://ipcd.ibis.ulaval.ca/index/display-detail/id/1370/criteria/IsolateID/dbfunction2/Y/
1374	SAMN10478528	RXBC00000000	SRR8612898	Germany	No	32	6.90149	501,065	66	31	https://ipcd.ibis.ulaval.ca/index/display-detail/id/1374/criteria/IsolateID/dbfunction2/Y/
1375	SAMN10478529	RXBB00000000	SRR8612893	Germany	Yes	44	6.79073	468,591	66.1	21	https://ipcd.ibis.ulaval.ca/index/display-detail/id/1375/criteria/IsolateID/dbfunction2/Y/
1376	SAMN10478530	RXBA00000000	SRR8612892	Germany	No	25	6.55867	546,946	66.2	31	https://ipcd.ibis.ulaval.ca/index/display-detail/id/1376/criteria/IsolateID/dbfunction2/Y/
1377	SAMN10478531	RXAZ00000000	SRR8612895	Germany	No	27	6.57869	777,188	66.4	29	https://ipcd.ibis.ulaval.ca/index/display-detail/id/1377/criteria/IsolateID/dbfunction2/Y/
1380	SAMN10478532	RXAY00000000	SRR8612894	France	No	23	6.23205	869,892	66.6	40	https://ipcd.ibis.ulaval.ca/index/display-detail/id/1380/criteria/IsolateID/dbfunction2/Y/
1381	SAMN10478533	RXAX00000000	SRR8612774	Austria	No	32	6.37824	471,841	66.4	25	https://ipcd.ibis.ulaval.ca/index/display-detail/id/1381/criteria/IsolateID/dbfunction2/Y/
1383	SAMN10478534	RXAW00000000	SRR8612773	Italy	No	20	6.38192	773,952	66.5	30	https://ipcd.ibis.ulaval.ca/index/display-detail/id/1383/criteria/IsolateID/dbfunction2/Y/
1384	SAMN10478535	RXAV00000000	SRR8612835	Sweden	No	25	6.17016	775,189	66.5	23	https://ipcd.ibis.ulaval.ca/index/display-detail/id/1384/criteria/IsolateID/dbfunction2/Y/
1385	SAMN10478536	RXAU00000000	SRR8612836	Germany	Yes	61	6.88093	200,292	66.1	21	https://ipcd.ibis.ulaval.ca/index/display-detail/id/1385/criteria/IsolateID/dbfunction2/Y/
1387	SAMN10478537	RXAT00000000	SRR8612837	Germany	Yes	48	7.2699	332,653	65.7	29	https://ipcd.ibis.ulaval.ca/index/display-detail/id/1387/criteria/IsolateID/dbfunction2/Y/
1389	SAMN10478538	RXAS00000000	SRR8612838	Germany	No	39	6.55809	511,900	66.3	29	https://ipcd.ibis.ulaval.ca/index/display-detail/id/1389/criteria/IsolateID/dbfunction2/Y/
1390	SAMN10478539	RXAR00000000	SRR8612831	Germany	No	58	6.64478	262,999	66.1	22	https://ipcd.ibis.ulaval.ca/index/display-detail/id/1390/criteria/IsolateID/dbfunction2/Y/
1396	SAMN10478540	RXAQ00000000	SRR8612832	Germany	No	48	6.40883	320,934	66.4	20	https://ipcd.ibis.ulaval.ca/index/display-detail/id/1396/criteria/IsolateID/dbfunction2/Y/
1399	SAMN10478541	RXAP00000000	SRR8612833	Germany	No	48	6.6459	264,812	66.1	41	https://ipcd.ibis.ulaval.ca/index/display-detail/id/1399/criteria/IsolateID/dbfunction2/Y/
1401	SAMN10478542	RXAO00000000	SRR8612834	Germany	No	36	6.44104	435,978	66.4	31	https://ipcd.ibis.ulaval.ca/index/display-detail/id/1401/criteria/IsolateID/dbfunction2/Y/
1405	SAMN10478543	RXAN00000000	SRR8612828	Germany	No	27	6.60307	781,951	66.1	30	https://ipcd.ibis.ulaval.ca/index/display-detail/id/1405/criteria/IsolateID/dbfunction2/Y/
1407	SAMN10478544	RXAM00000000	SRR8612829	Germany	No	34	6.5476	567,498	66.3	37	https://ipcd.ibis.ulaval.ca/index/display-detail/id/1407/criteria/IsolateID/dbfunction2/Y/
1408	SAMN10478545	RXAL00000000	SRR8612858	Germany	No	37	6.64896	375,085	66.2	33	https://ipcd.ibis.ulaval.ca/index/display-detail/id/1408/criteria/IsolateID/dbfunction2/Y/
1409	SAMN10478546	RXAK00000000	SRR8612857	Germany	No	91	6.57641	495,626	66.3	34	https://ipcd.ibis.ulaval.ca/index/display-detail/id/1409/criteria/IsolateID/dbfunction2/Y/
1412	SAMN10478547	RXAJ00000000	SRR8612856	Germany	No	25	6.47284	846,003	66.4	37	https://ipcd.ibis.ulaval.ca/index/display-detail/id/1412/criteria/IsolateID/dbfunction2/Y/
1413	SAMN10478548	RXAI00000000	SRR8612855	Germany	No	28	6.35215	538,741	66.5	34	https://ipcd.ibis.ulaval.ca/index/display-detail/id/1413/criteria/IsolateID/dbfunction2/Y/
1414	SAMN10478549	RXAH00000000	SRR8612862	Germany	No	79	7.01035	258,365	65.9	31	https://ipcd.ibis.ulaval.ca/index/display-detail/id/1414/criteria/IsolateID/dbfunction2/Y/
1415	SAMN10478550	RXAG00000000	SRR8612861	Germany	No	49	6.33354	395,233	66.3	34	https://ipcd.ibis.ulaval.ca/index/display-detail/id/1415/criteria/IsolateID/dbfunction2/Y/
1417	SAMN10478551	RXAF00000000	SRR8612860	Germany	No	23	6.37337	650,729	66.5	36	https://ipcd.ibis.ulaval.ca/index/display-detail/id/1417/criteria/IsolateID/dbfunction2/Y/
1419	SAMN10478552	RXAE00000000	SRR8612859	Germany	No	47	6.75195	451,143	66.1	27	https://ipcd.ibis.ulaval.ca/index/display-detail/id/1419/criteria/IsolateID/dbfunction2/Y/
1421	SAMN10478553	RXAD00000000	SRR8612854	Germany	No	25	6.45953	517,589	66.4	34	https://ipcd.ibis.ulaval.ca/index/display-detail/id/1421/criteria/IsolateID/dbfunction2/Y/
1422	SAMN10478554	RXAC00000000	SRR8612853	Germany	No	40	6.89816	461,251	66	26	https://ipcd.ibis.ulaval.ca/index/display-detail/id/1422/criteria/IsolateID/dbfunction2/Y/
1423	SAMN10478555	RXAB00000000	SRR8612790	Germany	No	25	6.42423	776,124	66.4	34	https://ipcd.ibis.ulaval.ca/index/display-detail/id/1423/criteria/IsolateID/dbfunction2/Y/
1427	SAMN10478556	RXAA00000000	SRR8612791	Germany	No	42	6.36034	282,058	66.4	33	https://ipcd.ibis.ulaval.ca/index/display-detail/id/1427/criteria/IsolateID/dbfunction2/Y/
1428	SAMN10478557	RWZZ00000000	SRR8612788	Germany	No	49	6.3626	386,090	66.4	32	https://ipcd.ibis.ulaval.ca/index/display-detail/id/1428/criteria/IsolateID/dbfunction2/Y/
1429	SAMN10478558	RWZY00000000	SRR8612789	Germany	No	20	6.43687	908,299	66.5	38	https://ipcd.ibis.ulaval.ca/index/display-detail/id/1429/criteria/IsolateID/dbfunction2/Y/
1432	SAMN10478559	RWZX00000000	SRR8612794	Germany	No	58	6.70811	261,267	66.1	30	https://ipcd.ibis.ulaval.ca/index/display-detail/id/1432/criteria/IsolateID/dbfunction2/Y/
1433	SAMN10478560	RWZW00000000	SRR8612795	Germany	No	42	6.37473	408,603	66.4	34	https://ipcd.ibis.ulaval.ca/index/display-detail/id/1433/criteria/IsolateID/dbfunction2/Y/
1434	SAMN10478561	RWZV00000000	SRR8612792	Germany	No	33	6.35399	490,002	66.5	33	https://ipcd.ibis.ulaval.ca/index/display-detail/id/1434/criteria/IsolateID/dbfunction2/Y/
1436	SAMN10478562	RWZU00000000	SRR8612793	Sweden	No	51	6.58658	451,797	66.3	29	https://ipcd.ibis.ulaval.ca/index/display-detail/id/1436/criteria/IsolateID/dbfunction2/Y/
1437	SAMN10478563	RWZT00000000	SRR8612786	Germany	No	33	6.3989	466,494	66.4	30	https://ipcd.ibis.ulaval.ca/index/display-detail/id/1437/criteria/IsolateID/dbfunction2/Y/
1438	SAMN10478564	RWZS00000000	SRR8612787	Germany	No	35	6.47752	448,595	66.3	30	https://ipcd.ibis.ulaval.ca/index/display-detail/id/1438/criteria/IsolateID/dbfunction2/Y/
1439	SAMN10478565	RWZR00000000	SRR8612803	Germany	No	41	6.3411	395,693	66.3	28	https://ipcd.ibis.ulaval.ca/index/display-detail/id/1439/criteria/IsolateID/dbfunction2/Y/
1615	SAMN10478566	RWZQ00000000	SRR8612802	USA	No	57	6.7142	437,944	66.3	34	https://ipcd.ibis.ulaval.ca/index/display-detail/id/1615/criteria/IsolateID/dbfunction2/Y/
1616	SAMN10478567	RWZP00000000	SRR8612805	USA	No	58	6.75495	227,745	66	35	https://ipcd.ibis.ulaval.ca/index/display-detail/id/1616/criteria/IsolateID/dbfunction2/Y/
1617	SAMN10478568	RWZO00000000	SRR8612804	USA	No	55	6.29945	286,961	66.5	46	https://ipcd.ibis.ulaval.ca/index/display-detail/id/1617/criteria/IsolateID/dbfunction2/Y/
1618	SAMN10478569	RWZN00000000	SRR8612807	USA	No	31	6.26749	776,900	66.5	47	https://ipcd.ibis.ulaval.ca/index/display-detail/id/1618/criteria/IsolateID/dbfunction2/Y/
1619	SAMN10478570	RWZM00000000	SRR8612806	USA	No	45	6.43522	421,454	66.3	35	https://ipcd.ibis.ulaval.ca/index/display-detail/id/1619/criteria/IsolateID/dbfunction2/Y/
1620	SAMN10478571	RWZL00000000	SRR8612809	USA	No	58	6.46408	257,773	66.3	33	https://ipcd.ibis.ulaval.ca/index/display-detail/id/1620/criteria/IsolateID/dbfunction2/Y/
1621	SAMN10478572	RWZK00000000	SRR8612808	USA	No	56	6.39597	392,325	66.3	42	https://ipcd.ibis.ulaval.ca/index/display-detail/id/1621/criteria/IsolateID/dbfunction2/Y/
1622	SAMN10478573	RWZJ00000000	SRR8612800	USA	No	45	6.28725	348,099	66.4	54	https://ipcd.ibis.ulaval.ca/index/display-detail/id/1622/criteria/IsolateID/dbfunction2/Y/
1623	SAMN10478574	RWZI00000000	SRR8612799	USA	No	64	6.76409	255,383	66.1	63	https://ipcd.ibis.ulaval.ca/index/display-detail/id/1623/criteria/IsolateID/dbfunction2/Y/
1624	SAMN10478575	RWZH00000000	SRR8612910	USA	No	38	6.38636	400,728	66.4	55	https://ipcd.ibis.ulaval.ca/index/display-detail/id/1624/criteria/IsolateID/dbfunction2/Y/

### Data availability.

Whole-genome shotgun projects have been deposited at DDBJ/EMBL/GenBank under accession numbers RWZH00000000 to RXFL00000000 as part of BioProject PRJNA325248. Details of the corresponding bacterial strain collection are available at https://ipcd.ibis.ulaval.ca/.
